# Sociodemographic variation in prescriptions dispensed in early pregnancy in Northern Ireland 2010–2016

**DOI:** 10.1371/journal.pone.0267710

**Published:** 2022-08-22

**Authors:** Joanne Given, Karen Casson, Helen Dolk, Maria Loane

**Affiliations:** Faculty of Life & Health Sciences, Ulster University, Belfast, Northern Ireland, United Kingdom; University of Oslo, NORWAY

## Abstract

**Aim:**

To establish the prevalence of prescriptions dispensed in early pregnancy by maternal age and area deprivation, for women who gave birth in Northern Ireland (NI) 2011–2016.

**Study design:**

Population-based linked cohort study.

**Methods:**

The NI Maternity System (NIMATS) database was used to identify all births to resident mothers in NI between 2011 and 2016. Prescriptions dispensed between the last menstrual period (LMP) and the first antenatal care visit (mean 10.7 weeks) (2010–2016) were extracted from the Enhanced Prescribing Database (EPD) which records all prescriptions dispensed by pharmacists in NI. EPD data were linked to NIMATS using the mother’s Health and Care Number. Maternal deprivation based on the NI Multiple Deprivation Measure 2017 was linked using the mother’s postcode.

**Results:**

The cohort included 139,687 pregnancies resulting in live or stillbirths to 106,206 women. A medication was dispensed in 63.5% of pregnancies, and in 48.7% of pregnancies excluding supplements (vitamins, iron, and folic acid). Folic acid was the most commonly dispensed medication (33.1%). Excluding supplements, the mean number of medications was 1.1, with 4.2% having ≥5 medications. The most common non-supplement medications were antibiotics (13.1%), antiemetics (8.7%), analgesics (6.9%), hormonal medications (6.9%) and antidepressants (6.1%). Younger women (<20 years) had more antibiotics while older women (40+ years) had more antidepressants, cardiovascular, antihypertensives, anticoagulant medications and thyroxine. The proportion of women living in the most deprived areas with prescriptions for antidepressants, sedatives, tranquilisers, analgesics, and anti-epileptic medications was double the proportion of women with these medications in the least deprived areas.

**Conclusion:**

Half of all pregnant women in NI were dispensed a non-supplement medication between LMP and the first antenatal care visit. Younger and older mothers and those living in the most deprived areas were more likely to have medications dispensed. More antidepressants were dispensed in areas of social deprivation.

## Introduction

Pregnant women often need to take medications to manage acute or chronic illnesses [1]. Despite this, there is insufficient information on the risks and safety for the vast majority of medications taken during pregnancy [2]. As few as 5% of available medications have been adequately monitored, tested and labelled with safety information for use in pregnant and breastfeeding women [3]. This is in part due to eligibility criteria for premarketing clinical trials of new medications which typically exclude high risk individuals such as pregnant women [4]. Monitoring of prescribing practices and further research into the safety of the most commonly prescribed medications is crucial to better understand risks and benefits to the fetus and the mother [5], optimise antenatal prescribing [6], assist in health care planning [7] and establish priorities for epidemiological research [6].

Use of prescription drugs during pregnancy varies dramatically across countries ranging from 27% in the United States to 93% in France [6]. Maternal age [8] and socioeconomic factors, such as education level [8, 9] and income [9, 10], have been found to be associated with medication use, and type of medication used, during pregnancy. The prevalence of medication use in pregnancy in Northern Ireland (NI), and how this varies with age and socioeconomic factors, is unknown. A potential source of information relating to medication use in pregnancy is routinely collected clinical/administrative data. There are two databases in NI which may be suitable for studying medication use in pregnancy but have not to date been linked for this purpose. The NI Maternity System (NIMATS) database captures all women giving birth in NI and the NI Enhanced Prescribing Database (EPD) includes all General Practitioner (GP) prescribed medications dispensed by pharmacists. These data can, in addition, be linked by postcode to area deprivation data.

Early pregnancy is a period of particular interest since it corresponds to organogenesis thus it is a period of fetal vulnerability. Also, in the first weeks of early pregnancy women may not know that they are pregnant, and they have not yet received advice about medication use which is given at the first antenatal visit. The aim of this study is to use linked administrative data to establish the prevalence of prescriptions dispensed between the last menstrual period (LMP) and the first antenatal visit, by maternal age and area deprivation, for women in NI who gave birth 2011–2016.

## Materials and methods

A cross-sectional data linkage study was conducted through the NI Business Services Organisation Honest Broker Service. The Honest Broker Service provides access within a secure environment to anonymised health and social care data for ethically approved research projects. The NIMATS database was used to identify all NI resident women who gave birth in NI between 1^st^ January 2011 and 31^st^ December 2016. Terminations of pregnancy were illegal in NI during the study period.

At the first visit for antenatal care, commonly known as the booking interview which usually occurs by week 10 [11], the midwife enters the woman’s demographic details, and reproductive and medical history in a bespoke database (NIMATS). A pregnancy resulting in multiple births (twins etc.) was counted only once in the dataset. For births in 2011, data on booking interviews were extracted from 2010. For births at the end of the 2016, information was extracted up to 30th June 2016 to cover exposure in early pregnancy.

Births with a gestational age at booking <1 week (n = 25) or a gestational age at delivery ≥44 weeks (n = 88) were excluded due to concerns about data validity. Those who booked very late > 20 weeks (n = 4,301, 3%) were also excluded as we were interested in prescriptions dispensed in early pregnancy.

For each pregnancy, the NIMATS record was linked to the EPD using the mother’s Health and Care Number (HCN) or hospital number if the HCN was unavailable. The information recorded in the EPD includes substance, generic and brand names, formulation, strength, quantity dispensed and date dispensed. All medications are classified using British National Formulary (BNF) codes with the addition of generic name or dose where necessary, S1 Table. A combination product such as an inhaler containing both an anti-asthmatic and a steroid is counted in both the anti-asthmatic and steroid categories, but it is only counted once when calculating the mean number of prescriptions. For each mother in NIMATS, all medications prescribed by a GP or Nurse (within a GP Practice) and dispensed by a community pharmacist for the period between the last menstrual period and the date of the booking interview were extracted from the EPD (calculated as the date of delivery minus the gestational age at delivery, to the date of the first antenatal visit). This exposure window was used as part of a study, to be reported separately, to compare prescriptions for medications recorded in the EPD to the medications reported to the midwife and recorded in NIMATS at the first antenatal visit. We set the upper limit of the exposure window as the date of the first antenatal appointment (up to and including 20 weeks) in order to capture early pregnancy exposures. In the NIMATS database, medications are recorded under one of 26 pre-defined categories including use of any vitamin, iron, or folic acid (hereafter referred to as supplements) and 22 categories of therapeutic medications, hence these medication categories were used in this study, see S1 Table for more information.

The NI Multiple Deprivation Measure 2017 (NIMDM) for the super output area in which the mother lived was linked using the mother’s postcode recorded in NIMATS at the first antenatal visit. The NIMDM is the official measure of deprivation in NI and ranks areas based on seven types or ‘domains’ of deprivation: Income Deprivation, Employment Deprivation, Health Deprivation and Disability, Education, Skills and Training Deprivation, Access to Services, Living Environment and Crime & Disorder [12, 13]. The overall NIMDM, composed from these domains, was used in this study recorded in quintiles (1 = most deprived, 5 = least deprived). Super output areas are geographical areas developed from Census 2001 information and are the lowest geographical area for which statistics were available. They are designed to have similar population sizes with populations as socially similar as possible. There are 890 super output areas in NI.

Descriptive statistics were used to describe the cohort. Prevalence of prescriptions dispensed in early pregnancy was defined as the number of pregnant women who had at least one prescription recorded in EPD divided by the total number of women giving birth, 2011–2016. The prevalence of medication by maternal age at the first antenatal visit (<20, 20–24, 25–29, 30–34, 35–39, 40+ years) and by deprivation quintiles were calculated for each of the medication groups recorded in NIMATS. Maternal age groups were combined when small numbers posed a perceived risk of disclosure as per Honest Broker Service policies. The Cochran-Armitage test for trend (chi-square test for trend and chi-square test for departure from linearity) [14] was used to identify trends in prescriptions dispensed over time, maternal age and NIMDM quintiles [15]. Stata/SE, version 14 (StataCorp. 2015) was used for all analyses.

This study was approved by Ulster University Nursing and Health Research Ethics Filter Committee, the Health Research Authority (reference: 17/NS/0047) and the Honest Broker Governance Board (HBS Reference 021). All data were anonymised before they were accessed and data were analysed anonymously so individual consent was not obtained.

## Results

The cohort consisted of 139,687 pregnancies, in 106,206 women. The mean gestational age at booking was 10.7 weeks with 50.3% of pregnancies booked within the recommended first ten weeks, and 86.3% of pregnancies booked by 12 gestational weeks. A small proportion (0.1%) had the first antenatal visit at <5 weeks gestational age. The mean gestational age at delivery was 39.0 weeks. 38.8% of pregnancies were first pregnancies. Mean maternal age was 29.3 years with most pregnancies (32.1%) occurring in the 30-34-year age group. 71.1% of pregnancies were planned with 26.9% unplanned. The majority of planned pregnancies were observed in mothers ≥25 years of age, see S2 Table, while the percentage of planned pregnancies was lowest in the most deprived areas compared to the least deprived areas, see S3 Table.

Between 2010 and 2016, 63.5% (88,693) of pregnancies had a medication recorded in the EPD, see Table 1. Excluding supplements, 48.7% (68,004) of pregnancies had a medication recorded.

**Table 1:** Number of women, number of pregnancies, and number, mean, and percentage of pregnancies with any medication and non-supplement medication dispensed from EPD.

	n
Number of women	106,206
Number of pregnancies	139,687
Mean gestational age at booking	10.7 weeks
Number of pregnancies with any medication	88,693 (63.5% of all pregnancies)
Mean number of any medication	1.4
Median (IQR)^a^ number of any medication	(0–2)
Number of pregnancies with non-supplement medication	68,004 (48.7% of all pregnancies)
Mean number of non supplement medications	1.1
Median (IQR) number of non supplement medication	0 (0–2)

^a^ IQR = Interquartile range.

One type of medication was dispensed for 28.6% of pregnancies, 15.5% had two, 8.5% had three, 4.8% had four and 6.1% had five or more medications dispensed. Excluding supplements, 22.5% of pregnancies had one medication dispensed, 12.1% had two, 6.4% had three, 3.4% had four and 4.2% had five or more recorded. The mean number of medications recorded was 1.4 (median 1, Inter quartile range (IQR) 0–2), which decreased to 1.1 (median 0, IQR 0–2) when supplements were excluded, see Table 1.

**Fig 1:**
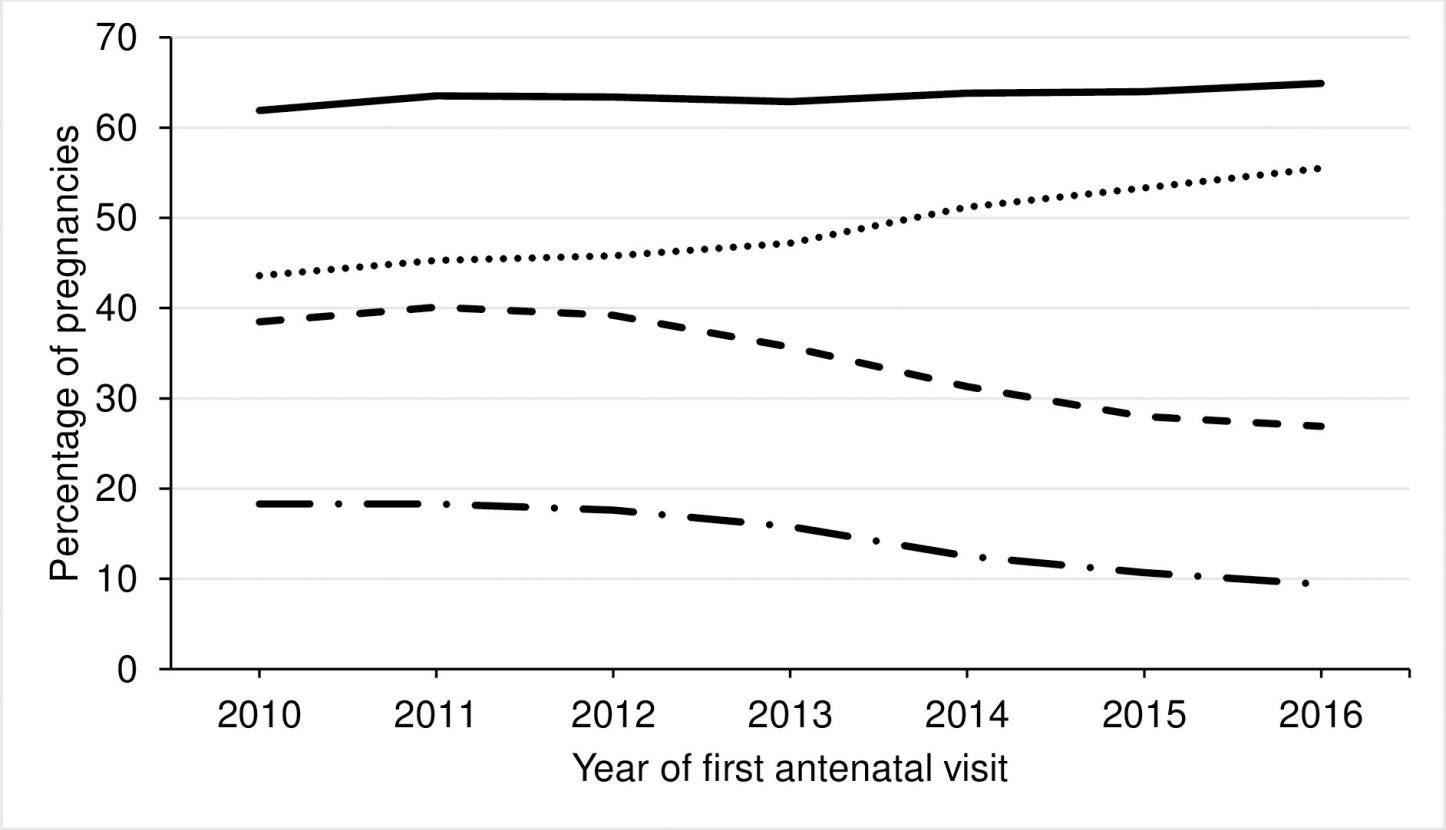
Percentage of pregnancies with any medication, non-supplement medication, any supplements (vitamins, iron or folic acid) and only supplements recorded by year of first antenatal visit. Solid black line = any medication; Dotted black line = non-supplement medication; Dashed black line = any supplements; Long dashed black line = only supplements.

The proportion of pregnant women with any medication recorded remained stable between 2010 and 2016. Prescriptions for non-supplements increased between 2010 (43.6%) and 2016 (55.5%) while any/only supplements decreased between 2012 and 2016, see Fig 1 and S4 Table. Non-linear trends were observed.

**Fig 2:**
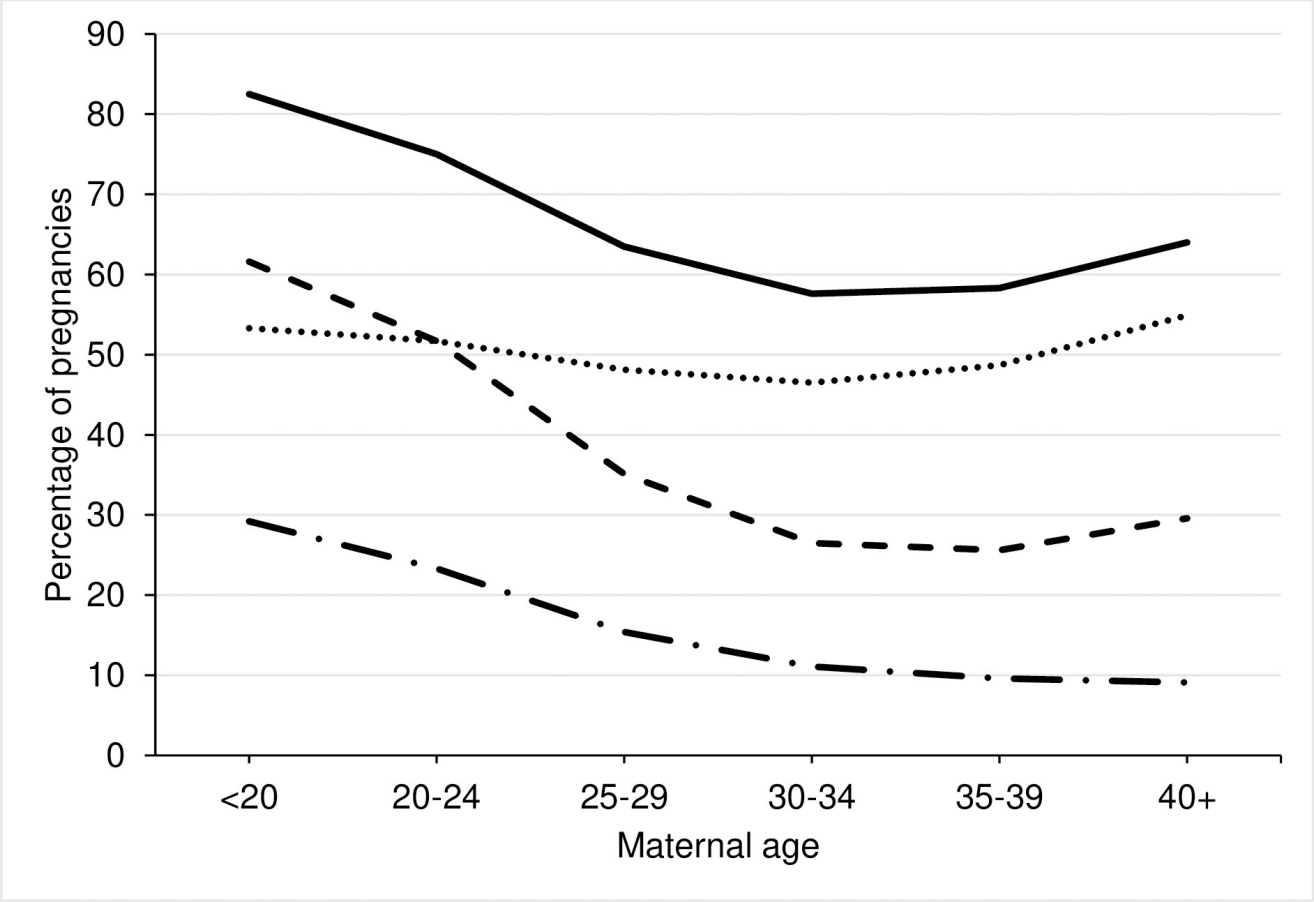
Percent of pregnancies with any medication, non-supplement medication, any supplements (vitamins, iron or folic acid) and only supplements recorded by maternal age. Solid black line = any medication; Dotted black line = non-supplement medication; Dashed black line = any supplements; Long dashed black line = only supplements.

Pregnant women <20 years were most likely to have a medication dispensed (82.5%), and of these, 35.4% were dispensed supplements only, see Fig 2 and S4 Table. The proportion of pregnant women with prescriptions for supplements only decreased with increasing age. When supplements were excluded, a greater proportion of mothers aged <20 years (53.3%) and mothers aged 40+ years (54.9%) compared to other maternal age groups had a medication dispensed in the EPD. Trends were non-linear.

**Fig 3:**
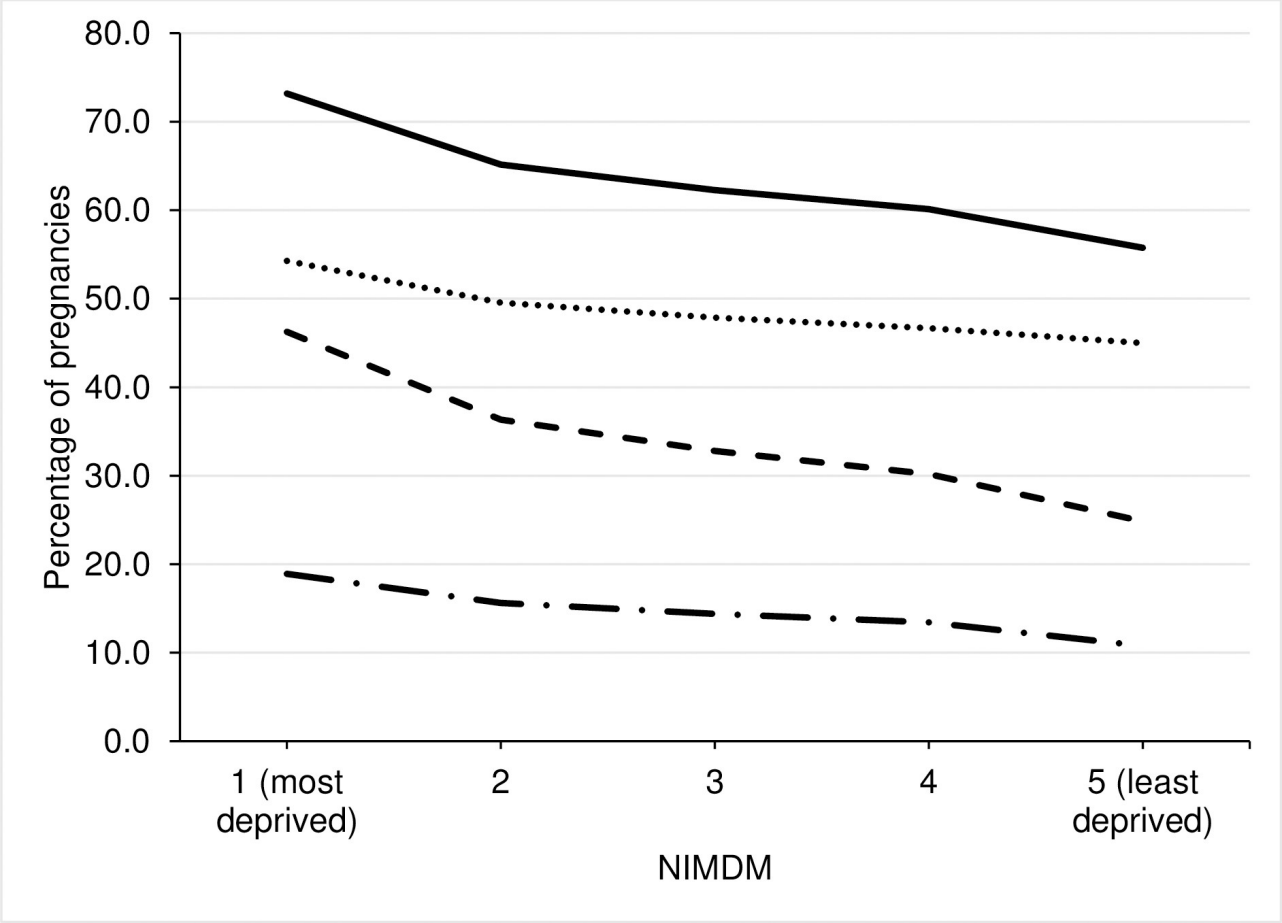
Percent of pregnancies with any medication, non-supplement medication, any supplements (vitamins, iron or folic acid) and only supplements recorded by NIMDM quintile. Solid black line = any medication; Dotted black line = non-supplement medication; Dashed black line = any supplements; Long dashed black line = only supplements.

Pregnant women living in the most deprived areas were most likely to have a medication dispensed and those living in the least deprived areas were least likely, see Fig 3 and S4 Table. This was true for all medications, those dispensed medications other than supplements and those dispensed only supplements (Fig 3) and S4 Table. Trends were non-linear.

### Maternal age and non-supplement medications

**Fig 4:**
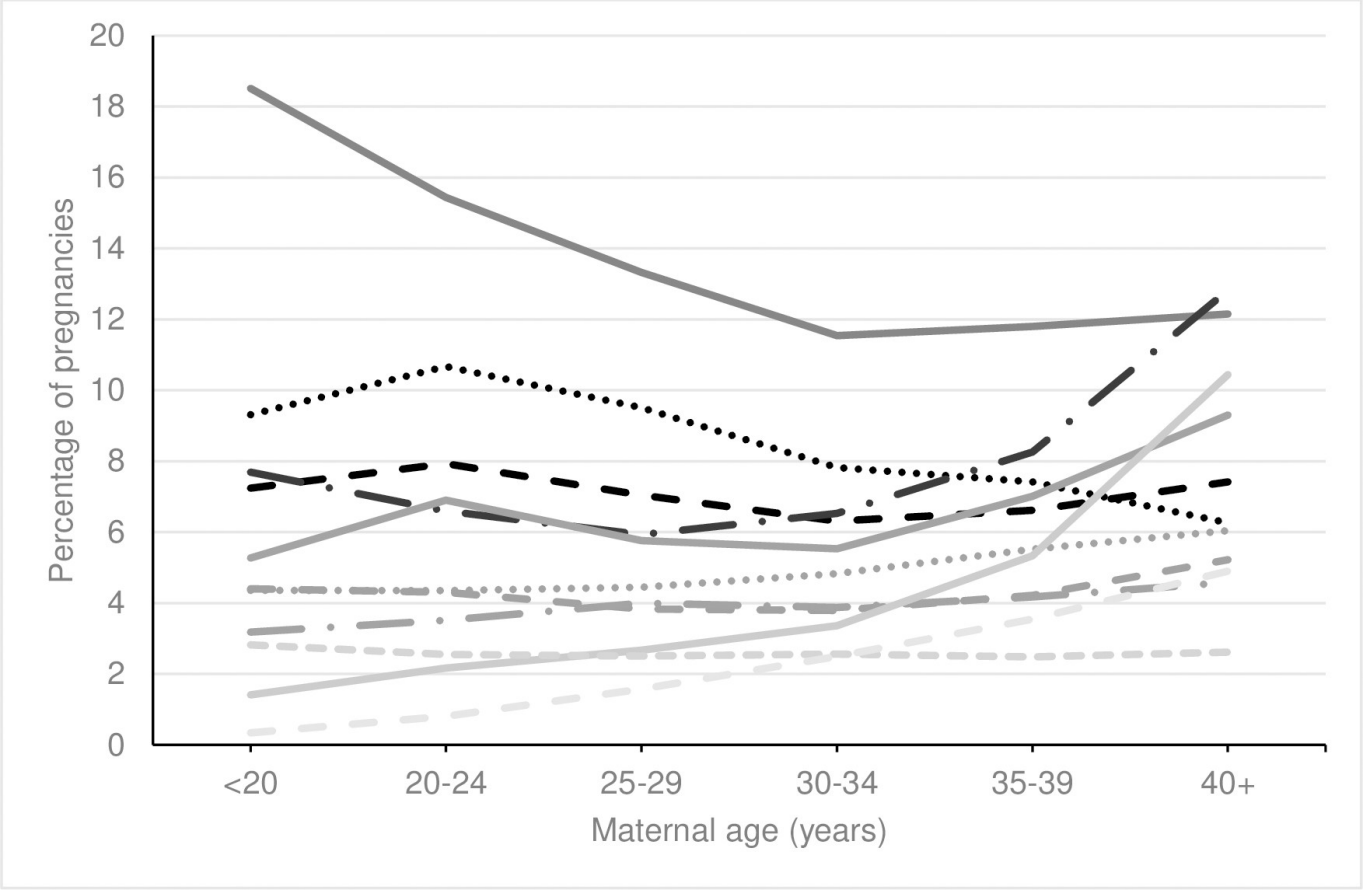
Percentage of pregnancies exposed to most common therapeutic classes of medication by maternal age. ^a^ Increasing trend (p<0.001). Solid black line = antibiotics; Dotted black line = antiemetics; Dashed black line = analgesics; Long dashed black line = hormonal medications; Solid grey line = antidepressants; Dotted gey line = steroids; Dashed grey line = antiasthmatics; Long dashed grey line = laxatives ^a^; Solid light grey line = cardiovascular medications; Dotted light grey line = antihistamines; Dashed light grey line = thyroxine.

**Fig 5:**
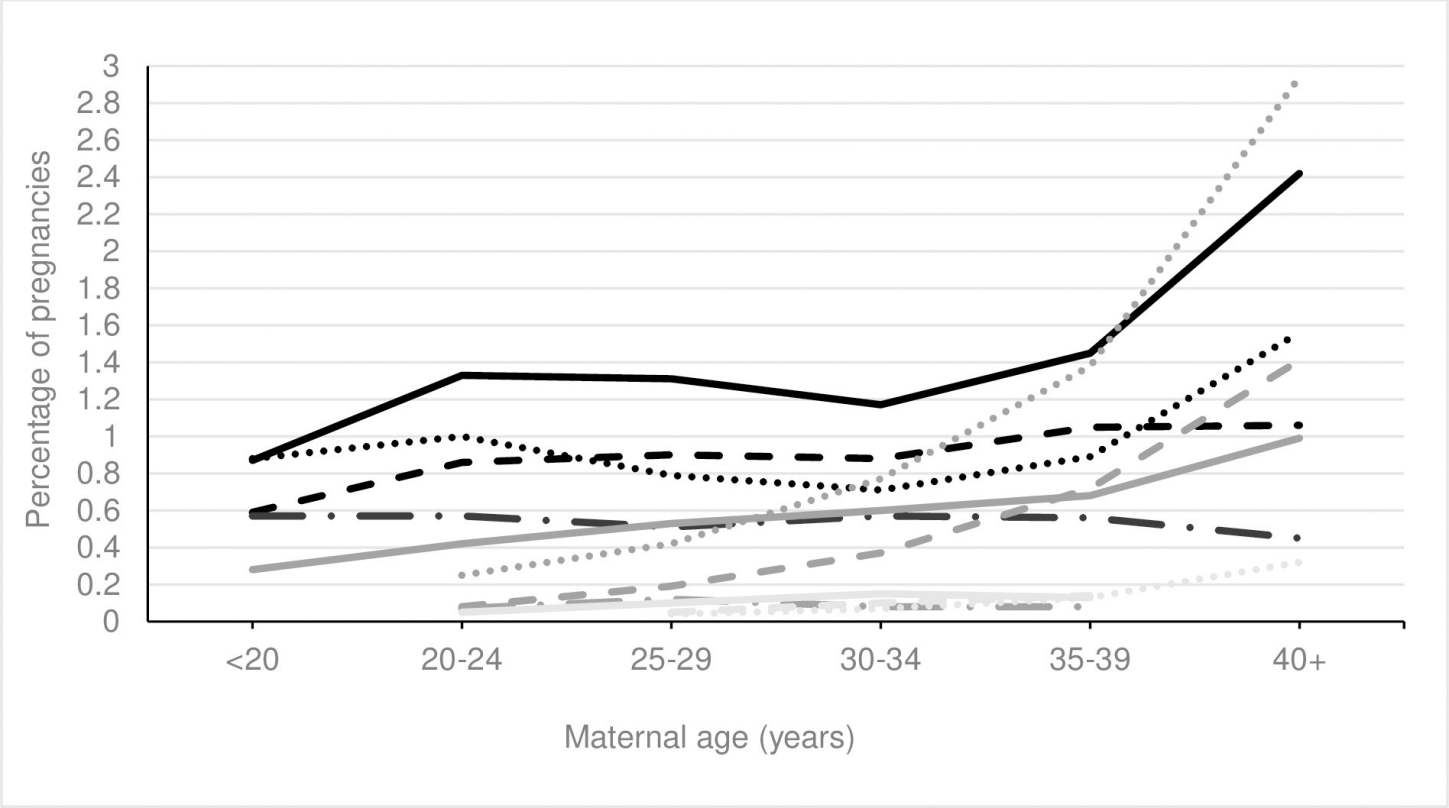
Percentage of pregnancies exposed to less common therapeutic classes of medication by maternal age. ^a^ Increasing trend p<0.001, ^b^ increasing trend p<0.01, ^c^ increasing trend p<0.05. Solid black line = tranquilizers; Dotted black line = sedatives; Dashed black line = antiepileptics ^b^; Long dashed black line = antivirals; Solid grey line = insulin ^a^; Dotted gey line = anticoagulants; Dashed grey line = antihypertensives; Long dashed grey line = antacids; Solid light grey line = immunosuppressants; Dotted light grey line = diuretics; Dashed light grey line = drug dependency ^c^.

When examining specific medication exposures, there was a statistically significantly increasing linear trend in the use of laxatives, antiepileptics, insulin and medication for alcohol or opioid dependence and a significantly decreasing linear trend in the use of antacids with increasing maternal age, see Figs 4 and 5 and S5 Table. No trend was seen between increasing maternal age and the use of anti asthmatics, antihistamines, sedatives, antivirals or immunosuppressants. A nonlinear trend was found for the remaining medications.

A greater proportion of younger pregnant women had folic acid recorded (<20 years 60.3%) compared to older pregnant women (40+ years 27.3%), see Figs 4 and 5 and S5 Table. Among the non-supplement medications, a higher proportion of younger pregnant women had antibiotics recorded (<20 years 18.5%) compared to older pregnant women (40+ years 12.1%). A higher proportion of older pregnant women had antidepressants (35–39 years, 7.0%, 40+ years 9.3%), cardiovascular (35–39 years 5.3%, 40+ years 10.4%), thyroxine (35–39 years 3.6%, 40+ years 4.9%), antihypertensive (35–39 years 0.7%, 40+ years 1.4%), and anticoagulant (35–39 years 1.4%, 40+ years 2.9%) medications recorded, see S3 Table.

### Social deprivation and non-supplement medications

**Fig 6:**
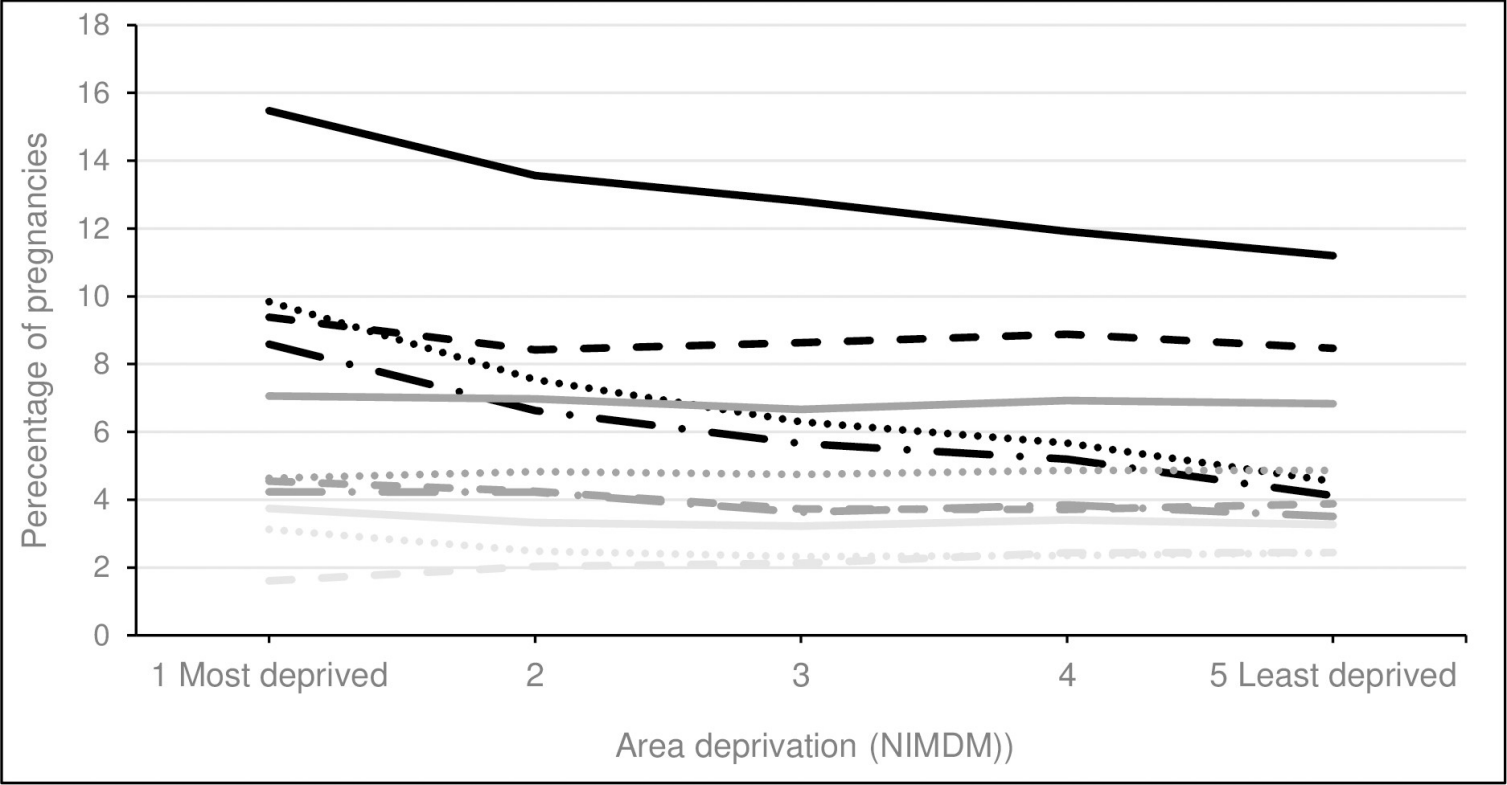
Percentage of pregnancies exposed to most common therapeutic classes of medication across area of deprivation (NIMDM). ^a^ Increasing trend p<0.001. Solid black line = antibiotics; Dotted black line = analgesics; Dashed black line = antiemetics; Long dashed black line = antidepressants; Solid grey line = hormonal medications; Dotted gey line = steroids; Dashed grey line = antiasthmatics; Long dashed grey line = laxatives; Solid light grey line = cardiovascular medications; Dotted light grey line = antihistamines; Dashed light grey line = thyroxine ^a^.

**Fig 7:**
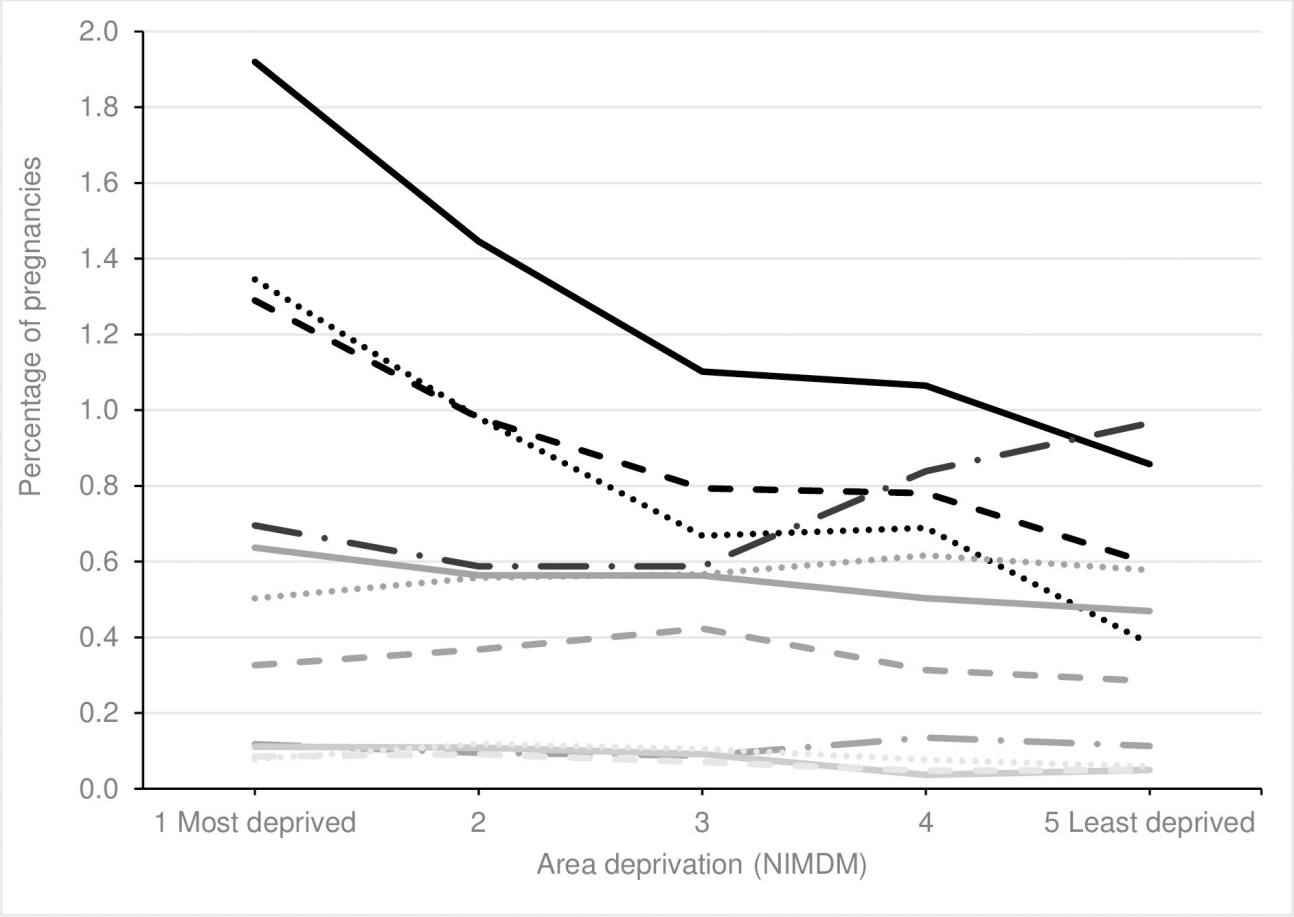
Percentage of pregnancies exposed to less common therapeutic classes of medication across area of deprivation (NIMDM). Solid black line = tranquilizers; Dotted black line = sedatives; Dashed black line = antiepileptics ^a^; Long dashed black line = anticoagulants; Solid grey line = antivirals ^b^; Dotted gey line = insulin; Dashed grey line = antihypertensives; Long dashed grey line = immunosuppressants; Solid light grey line = drug dependency^a^; Dotted light grey line = antacids; Dashed light grey line = diuretics ^c^.

We found a significantly increasing trend in the use of thyroxine and a significantly decreasing trend in the use of antiepileptics, antivirals, diuretics and medication for alcohol or opioid dependence with decreasing area-based deprivation, see Figs 6 and 7, S6 Table. No trend was seen between area deprivation and use of hormonal medications, steroids, insulin, antihypertensives, antacids and immunosuppressants. A non-linear trend was found for the remaining medications.

A greater proportion of pregnant women living in the most deprived areas at the first antenatal visit had supplements recorded in the EPD, mostly driven by post-conception folic acid. Pregnant women living in the most deprived areas generally also had higher proportions of non-supplement medications. The proportion of pregnant women living in the most deprived areas with prescriptions for antidepressants, sedatives, tranquilisers, analgesics, and anti-epileptic medications was double the proportion of women with these medications in the least deprived areas, see Figs 6 and 7, S6 Table.

## Discussion

In this large population-based study covering 139,687 pregnancies, almost two thirds had a prescribed medication dispensed between LMP and the first antenatal care visit. After excluding supplements, almost half were still dispensed medications. This is in in the lower range of early pregnancy exposures described in the literature where 57–99% of pregnancies have a medication recorded, 27–93% excluding vitamins and minerals [6].

The mean number of medications recorded, excluding supplements was 1.1 but 4% had five or more medications recorded. While for many drugs, there is little information on their safety in pregnancy [16, 17], there is even less information on the effects of combinations of medications during pregnancy [5]. Most research on prescription drug use during pregnancy examines a single therapeutic class and attempts to control for confounding by the underlying indication for the medicine [5]. From these results and other research exploring medication use in pregnancy, it appears to be relatively common for women to be treated for multiple conditions with multiple medications during pregnancy [5, 18–20].

The most commonly dispensed medication was folic acid with a third of pregnant women having folic acid prescribed and dispensed. It was possible to examine the dose of folic acid dispensed and just over a quarter of pregnant women had the recommended dose of 400 mcg preparation to prevent neural tube defects in women with low risk of conceiving a child with a neural tube defect [21]. Just over 6% of pregnancies had the higher 5mg dose dispensed which is recommended for women at a high risk of conceiving a child with a neural tube defect, including women who have previously had an infant with a neural tube defect, who are receiving antiepileptic medication, who have diabetes, sickle-cell disease [21–23] or who are obese [24]. Unlike low dose folic acid, which may be prescribed or bought over the counter, high dose folic acid can only be accessed through a prescription in NI. The prevalence of obesity/morbid obesity in pregnancy in NI is 19.8% [25]. High dose folic acid is, therefore, under prescribed, as allowing for the fact that a quarter of pregnancies were unplanned in our study, ~15% of pregnant women would be expected to be taking high dose folic acid due to their weight.

The five most frequently recorded non-supplement medications were antibiotics (13.1%), antiemetics (8.7%), analgesics (6.9%), hormonal medications (6.9%) and antidepressants (6.1%). Antibiotics, antiemetics and analgesics are frequently among the most prevalent medication exposures during pregnancy [5, 18–20, 26] and reflect how common urinary tract infections and nausea are during pregnancy. The hormonal medications include both the sex hormones, which may be given for pregnancy related indications, and the endocrine hormones including thyroxine and insulin, used to treat chronic illnesses. The prevalence of antidepressant use in NI pregnant women is high for a European country and more in keeping with that seen in the USA [27]. However, previous work examining mental health in NI has found a lifetime prevalence of mental disorders higher than any other Western European country [28]. This was also reflected in research on women of childbearing age in NI [29], which found that antidepressant use was high and increased with age and socioeconomic deprivation.

The youngest and oldest age groups had the largest proportion of pregnancies with a non-supplement medication recorded. The relatively high proportion of medication dispensed in the younger age group is due to use of antibiotics and analgesics while the older age groups were prescribed and dispensed more medications for chronic illnesses such as cardiovascular disease and depression. Given the trend for increasing mean maternal age [30, 31], the proportion of pregnancies exposed to these medications is likely to increase.

Those in the most socially deprived areas were most likely to have a medication dispensed and those in the least deprived areas were least likely. This is in keeping with greater medication use seen with low maternal education in Denmark [9] and Norway and low income in Denmark [9] and France [10]. A greater proportion of those living in the most deprived areas had folic acid dispensed. As low dose folic acid is available over the counter, this may be due to those women living in the most deprived areas requesting a prescription, which is free of charge, while those living in the least deprived areas buy folic acid. This is supported by previous work in England which found that mothers from higher social groups or with higher education were more likely to self-report use of folic acid [32] while younger women and those in more socioeconomically deprived areas were less likely [33]. Women buying medications over the counter may also explain why analgesics were dispensed more to women living in the most deprived areas compared to the least deprived areas.

Areas of greater deprivation had a higher use of antiepileptics, drugs for opioid dependence, and insulin, in keeping with what we know of the relationship of epilepsy [34, 35], substance abuse [36], diabetes and obesity [37] with socioeconomic deprivation and increased use of chronic/long-term medication in those with low education [8] and income [10]. Thyroxine was the only type of medication more common in the least deprived areas, possibly reflecting the higher average maternal age in the least deprived areas, or greater uptake of screening for thyroid conditions.

### Strengths and limitations

A major strength of the present study is the use of a population-based cohort comprising pregnancies to residents in NI during the study period.

The EPD contains all medications prescribed by a GP and dispensed by community pharmacies in NI. All prescriptions, including those issued on the advice of midwives at the first antenatal clinic, or at outpatients, are issued by GPs and then redeemed at community pharmacies. We included prescriptions dispensed up to an including the day of the first antenatal visit, which is unlikely to include prescriptions relating to that visit. This may result in underestimation of certain medications that are likely to be prescribed at maternity outpatients. There will also have been some degree of underreporting as 13.8% of prescriptions dispensed in primary care to the entire NI population, not just to pregnant women, could not be matched to a patient during the study period (from the data providers report on prescribing data quality). There was no information on drugs dispensed in hospital although those prescribed by specialists in the outpatient setting will usually be issued by the GP and be present in the data if issued before the first antenatal visit. There was also no information on medications which can be bought over the counter, such as analgesics, antihistamines, laxatives, and antacids, so the use of such medications will be underestimated. The sociodemographic pattern seen for such medications, which can be both prescribed and obtained over the counter, may be influenced by the ability to pay. Nevertheless, information on medication use during pregnancy derived directly from pharmacy dispensing databases is more accurate than patient recall, gross wholesale figures, or records of physician prescribing [7].

The EPD contains information on redeemed prescriptions and may overestimate medication exposure if women did not go on to take the medication they were dispensed [7]. While not equivalent to a measure of consumption by a patient, information on dispensed prescriptions is considered to be more accurate than data on issued prescriptions because, for a variety of reasons, many prescriptions written by doctors are not redeemed by patients [38]. In contrast to this, a study in the Netherlands reported a compliance rate of 84% for chronic medications and 92% of pregnancy-related conditions in the first trimester of pregnancy [39].

The variation in gestational age at booking means that medication use was not evaluated at a consistent time point in pregnancy. This may result in the recording of some medication exposures that do not reflect use in the first trimester. Also, as half of all booking appointments in this study occurred within the first ten weeks of pregnancy, the number of prescriptions dispensed up to the booking appointment may be underestimated when comparing with results concerning the entire first trimester. There is evidence suggesting that younger women (<20 years), women from ethnic minorities, and women from socially deprived areas are more likely to start their antenatal care later [40], hence excluding women who had their first antenatal appointment after 20 weeks may have slightly biased the overall figures for early pregnancy use.

The NIMDM is an area-based measure of deprivation. Not all those who live in the most deprived areas will be deprived and there may be people who are deprived living in the least deprived areas [13]. Nevertheless, this gives an ecological measure of the relationship between deprivation and dispensed prescriptions.

The number of comparisons made means that 2–3 significant linear trends would be expected as a result of chance alone.

This study was limited to describing patterns of medication prescribed and dispensed during pregnancy. It cannot evaluate the appropriateness of the medication prescribed for individual pregnant women.

## Conclusion

This is the first population-based study to describe medication prescribed and dispensed during pregnancy in NI. Half of all women were dispensed a non-supplement medication between LMP and the first antenatal care visit with those less than 25 years old, those 40+ years and those living in the most deprived areas most likely to have medication dispensed. The rate of dispensed antidepressants was high in areas of social deprivation.

## Supporting information

S1 Table BNF codes and generic drug names corresponding to the 26 medication categories recorded in the NIMATS system. (DOCX)

S2 Table Number and percentage of pregnancies planned by maternal age. (DOCX)

S3 Table Number and percentage of pregnancies planned by area of deprivation. (DOCX)

S4 Table Number and percentage of pregnancies with at least one medication recorded, no medication recorded, and total pregnancies by year of first antenatal visit, maternal age and NIMDM quintile. (DOCX)

S5 Table Number and percentage of pregnancies exposed to 26 therapeutic classes of medication by maternal age and trend with increasing maternal age. (DOCX)

S6 Table Number and percentage of pregnancies exposed to 26 therapeutic classes of medication by NIMDM quintiles and trend with decreasing area of deprivation. (DOCX)
