# Supplementary material for: Sociodemographic variation in prescriptions dispensed in early pregnancy in Northern Ireland 2010–2016
Source: PLoS One. 2022 Aug 22;17(8):e0267710. doi: 10.1371/journal.pone.0267710 (PMC9394805; doi:10.1371/journal.pone.0267710)
Supplement: S1 Table — (DOCX) [file pone.0267710.s001.docx]

S1 Table. BNF chapter and section codes and generic drug names corresponding to the 26 medication categories recorded in the NIMATS system

| **Medication** | **BNF chapter and section codes** | **BNF code and generic drug name^a^** |
| --- | --- | --- |
| **Supplements** | | |
| **Any vitamin, iron, or folic acid** | 9.6, 9.1.1 and 9.1.2 |  |
| **Folic acid (all doses)** | 9.1.2 |  |
| **Folic acid 400 mcg** | 9.1.2 with dose 400 mcg |  |
| **Folic acid 5mg** | 9.1.2 with dose 5mg |  |
| **Non-supplement medications** | | |
| **Antibiotics** | 5.1, 13.10.1 and 11.3.1 |  |
| **Antiemetics** | 4.6 |  |
| **Analgesics** | 4.7 |  |
| **Hormonal** | 6., 7.3, 8.3.1 and 8.3.2 |  |
| **Antidepressants** | 4.3 |  |
| **Steroids** | 1.5.2, 3.2, 6.3, 10.1.2, 11.4.1, 13.4 | 12.2.1 beclometasone, 12.2.1 betamethasone, 12.2.1 budesonide, 12.2.1 fluticasone, 12.2.1 mometasone, 12.2.1 triamcinolone, 12.3.1 betamethasone, 12.3.1 hydrocortisone |
| **Antiasthmatics** | 3.1, 3.2 and 3.3 |  |
| **Laxatives** | 1.6 |  |
| **Cardiovascular** | 2 | tadalafil |
| **Antihistamines** | 3.4.1 |  |
| **Thyroxine** | 6.2.1 |  |
| **Tranquilisers** | 4.1.2 |  |
| **Antiepileptics** | 4.8 |  |
| **Sedatives** | 4.1.1 |  |
| **Insulin** | 6.1.1 |  |
| **Antivirals** | 5.3, 13.10.3 and 11.3.3 |  |
| **Anticoagulants** | 2.8.1 and 2.8.2 |  |
| **Antihypertensives** | 2.5, 2.6.2 | tadalafil |
| **Antacids** | 1.1.1 |  |
| **Immunosuppressants** | 8.2.1, 8.2.2 and 1.5.3 |  |
| **Diuretics** | 2.2 |  |
| **Medication for alcohol or opioid dependence** | 4.10.1, 4.10.3 | acamprosate, disulfiram, calcium carbimide, nalmefene, buprenorphine, methadone, lofexidine, naltrexone, levacetylmethadol hydrochloride |

^a^ Some medications could not be identified by the BNF code alone and needed the generic drug name and BNF code to be combined, or generic drug name to be used, to identify exposures.
