# Supplementary material for: Sociodemographic variation in prescriptions dispensed in early pregnancy in Northern Ireland 2010–2016
Source: PLoS One. 2022 Aug 22;17(8):e0267710. doi: 10.1371/journal.pone.0267710 (PMC9394805; doi:10.1371/journal.pone.0267710)
Supplement: S2 Table — (DOCX) [file pone.0267710.s002.docx]

S2 Table. Number and percentage of pregnancies planned by maternal age

| **Pregnancy planning** | **Maternal age group** | | | | | | **All age groups**  **n (%)** |
| --- | --- | --- | --- | --- | --- | --- | --- |
|  | **<20**  **n (%)** | **20-24**  **n (%)** | **25-29**  **n (%)** | **30-34**  **n (%)** | **35-39**  **n (%)** | **40+**  **n (%)** |  |
| **Planned pregnancy** | 1,035 (16.0) | 10,167 (46.5) | 30,920 (75.6) | 37,125 (82.7) | 17,238 (80.1) | 2,810 (69.5) | 99,295 (71.1) |
| **Unplanned pregnancy** | 5,308 (82.3) | 11,290 (51.6) | 9,246 (23.0) | 6,812 (15.2) | 3,768 (17.5) | 1,107 (27.4) | 37,531 (26.9) |
| **Planning status unknown** | 109 (2.0) | 414 (2.0) | 731 (2.0) | 971 (2.2) | 511 (2.4) | 125 (3.1) | 2,861 (2.0) |
| **Total pregnancies** | 6,452 (4.6) | 21,871 (15.7) | 40,897 (29.3) | 44,908 (32.1) | 21,517 (15.4) | 4,042 (2.9) | 139687 |
