# Supplementary material for: Sociodemographic variation in prescriptions dispensed in early pregnancy in Northern Ireland 2010–2016
Source: PLoS One. 2022 Aug 22;17(8):e0267710. doi: 10.1371/journal.pone.0267710 (PMC9394805; doi:10.1371/journal.pone.0267710)
Supplement: S3 Table — (DOCX) [file pone.0267710.s003.docx]

S3 Table Number and percentage of pregnancies planned by area of deprivation (NIMDM)

| **Pregnancy planning** | **1 (Most deprived)**  **n (%)** | **2**  **n (%)** | **3**  **n (%)** | **4**  **n (%)** | **5 (Least deprived)**  **n (%)** | **Missing**  **n (%)** | **Total**  **n (%)** |
| --- | --- | --- | --- | --- | --- | --- | --- |
| **Planned pregnancy** | 18,279 (60) | 20,796 (70) | 21,047 (74) | 20,733 (76) | 17,457 (79) | 983 (0.7) | 99,295 (71.1) |
| **Unplanned pregnancy** | 12,000 (39) | 8,282 (28) | 7,036 (25) | 5,995 (22) | 4,078 (18) | 140 (0.1) | 37,531 (26.9) |
| **Planning status unknown** | 350  (1) | 540  (2) | 518 (2) | 697 (3) | 627  (3) | 129 (0.1) | 2,861 (2.0) |
| **Total pregnancies** | 30,629 (22) | 29,618  (21) | 28,601  (20) | 27,425  (20) | 22,162 (16) | 1,252 (1) | 139,687 |
