# Supplementary material for: Sociodemographic variation in prescriptions dispensed in early pregnancy in Northern Ireland 2010–2016
Source: PLoS One. 2022 Aug 22;17(8):e0267710. doi: 10.1371/journal.pone.0267710 (PMC9394805; doi:10.1371/journal.pone.0267710)
Supplement: S4 Table — (DOCX) [file pone.0267710.s004.docx]

S4 Table. Number and percentage of pregnancies with at least one medication recorded, no medication recorded, and total pregnancies by year of first antenatal visit, maternal age and NIMDM quintile

|  | | **At least one medication recorded** | | | | **Total pregnancies**  **n**  **(% total)** |
| --- | --- | --- | --- | --- | --- | --- |
|  |  | **Any medication**  **n**  **(%)** | **Non-supplement medication**  **n**  **(%)** | **Any supplements^a^**  **n**  **(%)** | **Only supplements**  **n**  **(%)** |  |
| **Year of first antenatal visit** | **2010** | 6,899  (61.9) | 4,861  (43.6) | 4,294  (38.5) | 2,038  (18.3) | 11,148  (8.0) |
|  | **2011** | 15,289  (63.5) | 10,889  (45.3) | 9,649  (40.1) | 4,400  (18.3) | 24,059  (17.2) |
|  | **2012** | 14,888  (63.4) | 10,757  (45.8) | 9,207  (39.2) | 4,131  (17.6) | 23,492  (16.8) |
|  | **2013** | 14,668  (62.9) | 10,996  (47.2) | 8,317  (35.7) | 3,672  (15.8) | 23,314  (16.7) |
|  | **2014** | 14,821  (63.8) | 11,910  (51.2) | 7,275  (31.3) | 2,911  (12.5) | 23,242  (16.6) |
|  | **2015** | 14,933  (64.0) | 12,438  (53.3) | 6,537  (28.0) | 2,495  (10.7) | 23,344  (16.7) |
|  | **2016** | 7,195  (64.9) | 6,153  (55.5) | 2,985  (26.9) | 1,042  (9.4) | 11,088  (7.9) |
| **Maternal age (completed years)** | **<20** | 5,321  (82.5) | 3,437  (53.3) | 3,973  (61.6) | 1,884  (29.2) | 6,452  (4.6) |
|  | **20-24** | 16,408  (75.0) | 11,312  (51.7) | 11,303  (51.7) | 5,096  (23.3) | 21,871  (15.7) |
|  | **25-29** | 25,957  (63.5) | 19,652  (48.1) | 14,361  (35.1) | 6,305  (15.4) | 40,897  (29.3) |
|  | **30-34** | 25,881  (57.6) | 20,904  (46.5) | 11,915  (26.5) | 4,977  (11.1) | 44,908  (32.1) |
|  | **35-39** | 12,538  (58.3) | 10,478  (48.7) | 5,515  (25.6) | 2,060  (9.6) | 21,517  (15.4) |
|  | **40+** | 2,588  (64.0) | 2,221  (54.9) | 1,197  (29.6) | 367  (9.1) | 4,042  (2.9) |
| **NIMDM Quintile** | **1 (most deprived)** | 22,413  (73.2) | 16,622  (54.3) | 14,168  (46.3) | 5,791  (18.9) | 30,629  (21.9) |
|  | **2** | 19,293  (65.1) | 14,671  (49.5) | 10,758  (36.3) | 4,622  (15.6) | 29,618  (21.2) |
|  | **3** | 17,810  (62.3) | 13,691  (47.9) | 9,384  (32.8) | 4,119  (14.4) | 28,601  (20.5) |
|  | **4** | 16,490  (60.1) | 12,803  (46.7) | 8,282  (30.2) | 3,687  (13.4) | 27,425  (19.6) |
|  | **5 (least deprived)** | 12,356  (55.8) | 9,969  (45.0) | 5,506  (24.8) | 2,387  (10.8) | 22,162  (15.9) |
|  | **Missing^b^** | 331  (26.4) | 248  (19.8) | 166  (13.3) | 83  (6.6) | 1,252  (0.9) |
| **Total** | | **88,693**  **(63.5)** | **68,004**  **(48.7)** | **48,264**  **(34.6)** | **20,689**  **(14.8)** | **139,687** |

^a^ Supplements= vitamins, iron, or folic acid; ^b^ No missing information for year of first antenatal visit or maternal age
