# Supplementary material for: Sociodemographic variation in prescriptions dispensed in early pregnancy in Northern Ireland 2010–2016
Source: PLoS One. 2022 Aug 22;17(8):e0267710. doi: 10.1371/journal.pone.0267710 (PMC9394805; doi:10.1371/journal.pone.0267710)
Supplement: S5 Table — (DOCX) [file pone.0267710.s005.docx]

S5 Table. Number and percentage of pregnancies exposed to 26 therapeutic classes of medication by maternal age and trend with increasing maternal age

| **Medication** | **Total exposed n (%)** | **Maternal age group (years)** | | | | | | | | | | | | | | **Test for trend**  **(p value^1^)** |
| --- | --- | --- | --- | --- | --- | --- | --- | --- | --- | --- | --- | --- | --- | --- | --- | --- |
|  |  | **<20**  **n (%)** | **20-24**  **n (%)** | | | **25-29**  **n (%)** | | | **30-34**  **n (%)** | | **35-39**  **n (%)** | | | **40+**  **n (%)** | |  |
| **Supplements** | | | | | | | | | | | | | | | | |
| **Any vitamin, iron, or folic acid** | 48,264 (34.6) | 3,973 (61.6) | 11,303 (51.7) | | | 14,361 (35.1) | | | 11,915 (26.5) | | 5,515 (25.6) | | | 1,197 (29.6) | | Non-linear trend |
| **Folic acid post-conception (all doses)** | 46,223 (33.1) | 3,889 (60.3) | 11,029 (50.4) | | | 13,827 (33.8) | | | 11,229 (25.0) | | 5,147 (23.9) | | | 1,102 (27.3) | | Non-linear trend |
| **Folic acid 400 mcg post-conception** | 37,654 (27.0) | 3,889 (54.2) | 11,029 (44.4) | | | 13,827 (28.0) | | | 11,229 (19.2) | | 5,147 (17.0) | | | 1,102 (17.5) | | Non-linear trend |
| **Folic acid 5mg post-conception** | 8,985 (6.4) | 426 (6.6) | 1,431 (6.5) | | | 2,485 (6.1) | | | 2,685 (6.0) | | 1,546 (7.2) | | | 412 (10.2) | | Non-linear trend |
| **Non-supplement medications** | | | | | | | | | | | | | | | | |
| **Antibiotics** | 18,230 (13.1) | 1,194 (18.5) | 3,377 (15.4) | | | 5,446 (13.3) | | | 5,182 (11.5) | | 2,540 (11.8) | | | 491 (12.1) | | Non-linear trend |
| **Antiemetics** | 12,187 (8.7) | 601 (9.3) | 2,334 (10.7) | | | 3,890 (9.5) | | | 3,512 (7.8) | | 1,597 (7.4) | | | 253 (6.3) | | Non-linear trend |
| **Analgesics** | 9,640 (6.9) | 467 (7.2) | 1,735 (7.9) | | | 2,881 (7.0) | | | 2,832 (6.3) | | 1,425 (6.6) | | | 300 (7.4) | | Non-linear trend |
| **Hormonal^2^** | 9,592 (6.9) | 496 (7.7) | 1,442 (6.6) | | | 2,427 (5.9) | | | 2,932 (6.5) | | 1,777 (8.3) | | | 518 (12.8) | | Non-linear trend |
| **Antidepressants** | 8,574 (6.1) | 340 (5.3) | 1,509 (6.9) | | | 2,356 (5.8) | | | 2,484 (5.5) | | 1,509 (7.0) | | | 376 (9.3) | | Non-linear trend |
| **Steroids** | 6,647 (4.8) | 281 (4.4) | 951 (4.3) | | | 1,816 (4.4) | | | 2,167 (4.8) | | 1,188 (5.5) | | | 244 (6.0) | | Non-linear trend |
| **Antiasthmatics** | 5,614 (4.0) | 284 (4.4) | 941 (4.3) | | | 1,567 (3.8) | | | 1,702 (3.8) | | 909 (4.2) | | | 211 (5.2) | | No trend |
| **Laxatives** | 5,431 (3.9) | 205 (3.2) | 767 (3.5) | | | 1,636 (4.0) | | | 1,741 (3.9) | | 897 (4.2) | | | 185 (4.6) | | Increasing (<0.001) |
| **Cardiovascular^3^** | 4,733 (3.4) | 91 (1.4) | 474 (2.2) | | | 1,091 (2.7) | | | 1,508 (3.4) | | 1,147 (5.3) | | | 422 (10.4) | | Non- linear trend |
| **Antihistamines** | 3,554 (2.5) | 182 (2.8) | 558 (2.6) | | | 1,026 (2.5) | | | 1,148 (2.6) | | 534 (2.5) | | | 106 (2.6) | | No trend |
| **Thyroxine** | 2,926 (2.1) | 22 (0.3) | 178 (0.8) | | | 644 (1.6) | | | 1,120 (2.5) | | 764 (3.6) | | | 198 (4.9) | | Non-linear trend |
| **Tranquilisers** | 1,818 (1.3) | 56 (0.9) | 291 (1.3) | | | 534 (1.3) | | | 527 (1.2) | | 312 (1.5) | | | 98 (2.4) | | Non-linear trend |
| **Antiepileptics** | 1,261 (0.9) | 38 (0.6) | 189 (0.9) | | | 368 (0.9) | | | 396 (0.9) | | 227 (1.1) | | | 43 (1.1) | | Increasing (0.002) |
| **Sedatives** | 1,170 (0.8) | 57 (0.9) | 219 (1.0) | | | 322 (0.8) | | | 318 (0.7) | | 191 (0.9) | | | 63 (1.6) | | No trend |
| **Insulin** | 782 (0.6) | 18 (0.3) | 92 (0.4) | | | 215 (0.5) | | | 270 (0.6) | | 147 (0.7) | | | 40 (1.0) | | Increasing (<0.001) |
| **Antivirals** | 766 (0.5) | 37 (0.6) | 125 (0.6) | | | 209 (0.5) | | | 257 (0.6) | | 120 (0.6) | | | 18 (0.4) | | No trend |
|  |  | **<25**  **n (%)** | | **25-29**  **n (%)** | | | | **30-34**  **n (%)** | | **35-39**  **n (%)** | | | | | **40+**  **n (%)** |  |
| **Anticoagulants** | 1,002 (0.7) | 70 (0.2) | | 173 (0.4) | | | | 344 (0.8) | | 296 (1.4) | | | | | 119 (2.9) | Non-linear trend |
| **Antihypertensives** | 480 (0.3) | 24 (0.1) | | 79 (0.2) | | | | 166 (0.4) | | 154 (0.7) | | | | | 57 (1.4) | Non-linear trend |
|  |  | **<25**  **n (%)** | | | **25-29**  **n (%)** | | | | **30-34**  **n (%)** | | | | **35+**  **n (%)** | | |  |
| **Antacids** | 124 (0.1) | 21 (0.1) | | | 48 (0.1) | | | | 35 (0.1) | | | | 20 (0.1) | | | Decreasing  (0.002) |
| **Immunosuppressants** | 152 (0.1) | 13 (0.0) | | | 39 (0.1) | | | | 66 (0.1) | | | | 34 (0.1) | | | No trend |
|  |  | **<30**  **n (%)** | | | **30-34**  **n (%)** | | | | **35-39**  **n (%)** | | | | **40+**  **n (%)** | | |  |
| **Diuretics** | 97 (0.1) | 27 (0.0) | | | 30 (0.1) | | | | 27 (0.1) | | | | 13 (0.3) | | | Non-linear trend |
|  |  | **<30**  **n (%)** | | | | | **30-34**  **n (%)** | | | | | **35+**  **n (%)** | | | |  |
| **Medication for alcohol or opioid dependence** | 113 (0.1) | 32 (0.0) | | | | | 46 (0.1) | | | | | 35 (0.1) | | | | Increasing (0.02) |
| ^1^ Chi-square test for trend. Given for linear trends only. The Chi-square test for departure from linearity will be non-significant (not provided).  ^2^ Includes endocrine system, contraceptives, oestrogens, and progestogens.  ^3^ Includes positive inotropic drugs, diuretics, anti-arrhythmic drugs, beta-adrenoceptor blocking drugs, hypertension and heart failure, nitrates, calcium-channel blockers & other antianginal drugs, sympathomimetics, anticoagulants and protamine, antiplatelet drugs, stable angina, acute coronary syndromes, and fibrinolysis, antifibrinolytic drugs and haemostatics, lipid-regulating drugs and local sclerosants. | | | | | | | | | | | | | | | | |
