# Supplementary material for: Sociodemographic variation in prescriptions dispensed in early pregnancy in Northern Ireland 2010–2016
Source: PLoS One. 2022 Aug 22;17(8):e0267710. doi: 10.1371/journal.pone.0267710 (PMC9394805; doi:10.1371/journal.pone.0267710)
Supplement: S6 Table — (DOCX) [file pone.0267710.s006.docx]

S6 Table. Number and percentage of pregnancies exposed to 26 therapeutic classes of medication by NIMDM quintiles and trend with decreasing area of deprivation

| **Medication** | **NIMDM quintile** | | | | | | **Test for trend**  **(p value^1^)** |
| --- | --- | --- | --- | --- | --- | --- | --- |
|  | **1 (most deprived)**  **n (%)** | **2**  **n (%)** | **3**  **n (%)** | **4**  **n (%)** | **5 (least deprived)**  **n (%)** | **Missing**  **n (%)** |  |
| **Supplements** | | | | | | | |
| **Any vitamins, iron, or folic acid** | 14,168 (46.3) | 10,758 (36.3) | 9,384 (32.8) | 8,282 (30.2) | 5,506 (24.8) | 166 (13.3) | Non-linear trend |
| **Folic acid post-conception (all doses)** | 13,678 (44.7) | 10,281 (34.7) | 8,944 (31.3) | 7,925 (28.9) | 5,232 (23.6) | 163 (13.0) | Non-linear trend |
| **Folic 400 mcg post-conception** | 11,439 (37.3) | 8,503 (28.7) | 7,263 (25.4) | 6,338 (23.1) | 3,977 (17.9) | 134 (10.7) | Non-linear trend |
| **Folic 5mg post-conception** | 2,397 (7.8) | 1,884 (6.4) | 1,745 (6.1) | 1,643 (6.0) | 1,286 (5.8) | 30 (2.4) | Non-linear trend |
| **Non-supplements** | | | | | | | |
| **Antibiotics** | 4,740 (15.5) | 4,018 (13.6) | 3,661 (12.8) | 3,268 (11.9) | 2,482 (11.2) | 61 (4.9) | Non-linear trend |
| **Antiemetics** | 2,874 (9.4) | 2,494 (8.4) | 2,470 (8.6) | 2,436 (8.9) | 1,876 (8.5) | 37 (3.0) | Non-linear trend |
| **Analgesics** | 3,014 (9.8) | 2,235 (7.5) | 1,801 (6.3) | 1,554 (5.7) | 1,006 (4.5) | 30 (2.4) | Non-linear trend |
| **Hormonal^2^** | 2,162 (7.1) | 2,065 (7.0) | 1,906 (6.7) | 1,898 (6.9) | 1,513 (6.8) | 48 (3.8) | No trend |
| **Antidepressants** | 2,630 (8.6) | 1,964 (6.6) | 1,617 (5.7) | 1,425 (5.2) | 913 (4.1) | 25 (2.0) | Non-linear trend |
| **Steroids** | 1,419 (4.6) | 1,431 (4.8) | 1,356 (4.7) | 1,333 (4.9) | 1,078 (4.9) | 30 (2.4) | No trend |
| **Antiasthmatics** | 1,393 (4.5) | 1,257 (4.2) | 1,066 (3.7) | 1,018 (3.7) | 861 (3.9) | 19 (1.5) | Non-linear trend |
| **Laxatives** | 1,296 (4.2) | 1,252 (4.2) | 1,038 (3.6) | 1,053 (3.8) | 777 (3.5) | 15 (1.2) | Non-linear trend |
| **Cardiovascular^3^** | 1,146 (3.7) | 985 (3.3) | 921 (3.2) | 933 (3.4) | 724 (3.3) | 24 (1.9) | Non-linear trend |
| **Antihistamines** | 957 (3.1) | 736 (2.5) | 664 (2.3) | 646 (2.4) | 540 (2.4) | 11 (0.9) | Non-linear trend |
| **Thyroxine** | 492 (1.6) | 601 (2.0) | 608 (2.1) | 669 (2.4) | 541 (2.4) | 15 (1.2) | Increasing (<0.001) |
| **Tranquilizers** | 588 (1.9) | 428 (1.4) | 315 (1.1) | 292 (1.1) | 190 (0.9) | <10 | Non-linear trend |
| **Antiepileptics** | 395 (1.3) | 290 (1.0) | 227 (0.8) | 214 (0.8) | 132 (0.6) | <10 | Decreasing  (<0.001) |
| **Sedatives** | 412 (1.3) | 290 (1.0) | 191 (0.7) | 189 (0.7) | 85 (0.4) | <10 | Non-linear trend |
| **Insulin** | 154 (0.5) | 165 (0.6) | 162 (0.6) | 169 (0.6) | 128 (0.6) | <10 | No trend |
| **Antivirals** | 195 (0.6) | 167 (0.6) | 161 (0.6) | 138 (0.5) | 104 (0.5) | <10 | Decreasing  (0.006) |
| **Anticoagulants** | 213 (0.7) | 174 (0.6) | 168 (0.6) | 230 (0.8) | 214 (1.0) | <10 | Non-linear trend |
| **Antihypertensives** | 100 (0.3) | 109 (0.4) | 121 (0.4) | 86 (0.3) | 63 (0.3) | <10 | No trend |
| **Antacids** | 24 (0.1) | 35 (0.1) | 30 (0.1) | 21 (0.1) | 13 (0.1) | <10 | No trend |
| **Immunosuppressants** | 36 (0.1) | 28 (0.1) | 12 (0.0) | 37 (0.1) | 13 (0.1) | 26 (2.1) | No trend |
| **Diuretics** | 26 (0.1) | 27 (0.1) | 20 (0.1) | 13 (0.0) | 11 (0.0) | <10 | Decreasing  (0.02) |
| **Medication for alcohol or opioid dependence** | 34 (0.1) | 32 (0.1) | 26 (0.1) | 10 (0.0) | 11 (0.0) | <10 | Decreasing (<0.001) |
| ^1^ Chi-square test for trend. Given for linear trends only. The Chi-square test for departure from linearity will be non-significant (not provided).  ^2^ Includes endocrine system, contraceptives, oestrogens, and progestogens.  ^3^ Includes positive inotropic drugs, diuretics, anti-arrhythmic drugs, beta-adrenoceptor blocking drugs, hypertension and heart failure, nitrates, calcium-channel blockers & other antianginal drugs, sympathomimetics, anticoagulants and protamine, antiplatelet drugs, stable angina, acute coronary syndromes, and fibrinolysis, antifibrinolytic drugs and haemostatics, lipid-regulating drugs and local sclerosants. | | | | | | | |
